# Knockdown of *stat3* expression by RNAi inhibits *in vitro* growth of human ovarian cancer

**DOI:** 10.2478/v10019-011-0013-8

**Published:** 2011-04-28

**Authors:** Shu-Hua Zhao, Fan Zhao, Jing-Ying Zheng, Li-Fang Gao, Xue-Jian Zhao, Man-Hua Cui

**Affiliations:** 1 Second Clinical Hospital of Jilin University, Changchun, China; 2 Department of Pathophysiology, School of Basic Medical Sciences, Jilin University, Changchun, China

**Keywords:** RNA interference, *stat3*, SKOV3 cells, ovarian cancer, apoptosis

## Abstract

**Background:**

The aim of the study was to investigate the suppressive effects of pSilencer2.1-U6-siRNA-*stat3* recombinant plasmids on the growth of ovarian cancer *in vitro*.

**Material and methods.:**

Three pairs of DNA template (*stat3*-1, *stat3*-2, *stat3*-3) specific for different target sites on *stat3* mRNA were synthesized to reconstruct pSilencer2.1-U6-siRNA-*stat3*s, which were transfected into SKOV3 cells. The expressions of STAT3, BcL-2, cyclin D1 and C-myc in these cells were detected by Western blot and Northern blot. The cell cycle and the growth were determined by flow cytometry (FCM) and MTT assay, respectively. Cell apoptosis was determined by TUNEL staining.

**Results:**

Of the three siRNAs, only siRNA targeting *stat3*-3 markedly suppressed the protein expression of *stat3* in SKOV3 cells; MTT assay and FCM showed that transfection of *stat3*-3 siRNA could significantly suppress the growth of SKOV3 cells and arrest the cell cycle *in vitro*. TUNEL staining also showed massive apoptosis in SKOV3 cells transfected with *stat3*-3 siRNA.

**Conclusions:**

pSilencer2.1-U6-siRNA-*stat3*-3 can significantly inhibit the STAT3 expression in human ovarian cancer cells resulting in the inhibition of the cancer growth and the increase of apoptosis of cancer cells.

## Introduction

Ovarian cancer, such as serous carcinoma, mucinous carcinoma, endometrioid carcinoma, metastatic carcinoma etc., is one of the common gynecological malignant tumors with high mortality. It is highly malignant and its incidence is increasing.^1^ Although great advances have been achieved in the radiotherapy and chemotherapy of ovarian cancer, the 5-year survival rate of ovarian cancer is not improved significantly.^2^ Further understanding of the molecular mechanisms underlying the proliferation, differentiation and survival of gynecological cancer cells is critical for the development of optimal therapeutic modalities. Although the molecular changes in the development of ovarian cancer are not completely understood, a lot of genes have been found to be involved in the occurrence and development of gynecological cancers.^3^,^4^ Thus, these genes may be useful targets in the development of specific antitumor therapeutic strategies. Studies have demonstrated the signal transducers and activators of transcription 3 (STAT3) signaling pathway plays a key role in the carcinogenesis by promotion of proliferation, differentiation and cell cycle progression, as well as the inhibition of apoptosis.^5^,^6^ The constitutive activation of STAT3 is implicated in a variety of cancer cell lines^7^–^11^, which suggests *stat3* may be an important molecular target for the anti-tumor therapy. Recent studies demonstrate the blockade of STAT3 expression in human cancer cells that suppresses the *in vitro* proliferation of cancer cells and the *in vivo* tumorigenicity. Attempts to suppress STAT3 expression have been made using tyrosine kinase inhibitors^12^,^13^, antisense oligonucleotides^14^, decoy oligonucleotides^15^, dominant-negative STAT3 protein^16^,^17^ and RNA interference (RNAi).^18^,^19^ *In vitro* studies have shown that the inhibition of STAT3 activity in human cancer cells can induce the apoptosis and/or the growth arrest. In human head and neck squamous carcinoma cells, prostate cancer cells, and laryngeal cancer cells, blocking of *stat3* by decoy oligonucleotides or antisense oligonucleotides or siRNA abrogates the production of transforming growth factor (TGF) and suppresses the oncogenic growth of these cells.^15^ Furthermore, some studies have revealed some apoptosis-related genes, such as Bcl-xL, C-myc and cyclin D1, etc, that are involved in the STAT3 blockage induced suppression of cancer cell growth.

RNAi is triggered by the presence of double-stranded RNA (dsRNA) in cells and results in rapid degradation of targeted mRNA with homology to double strand RNA leading to potent and selective silencing of genes. RNAi provides a novel approach to inhibit gene expression, and to date, RNAi with siRNA has been applied as a functional genomic tool.^20^

In the present study, siRNA targeting *stat3* gene was synthesized and siRNA-*stat3* expression vectors were constructed with pSilencer 2.1-U6, which was then used to transfect human ovarian cancer cells (SKOV3 cells) aiming to inhibit the STAT3 expression and induce apoptosis of cancer cells. SKOV3 cells transfected with siRNA-*stat3* were subcutaneously injected into nude mice and the growth and the apoptosis of ovarian cancer cells were observed.

## Materials and methods

### Immunohistochemistry for stat3 in the ovarian cancer

Twenty-five ovarian cancer samples and twenty fresh normal ovary tissues were collected for the determination of STAT3 expression. These tissues were embedded in paraffin and cut into 5-μm sections. After deparaffinization, the endogenous peroxidase was inactivated by 3% hydrogen peroxide in methanol for 10 min. The sections were treated with rabbit anti-human STAT3 polyclonal antibody (Santa Cruz, USA) and then with goat anti-rabbit IgG conjugated with horseradish peroxidase. The development was done at room temperature, using an avidin-biotin-peroxidase complex method (Vectastain Elite ABC kit; Vector Laboratories). The criteria for grading of STAT3 expression were as follows: negative (−): ≤5% positive cells; low (+): 5∼25% positive cells; moderate (++): 25∼50% positive cells; strong (+++): 50∼100% positive cells).

### Plasmid construction and determination

Three pairs of double stranded siRNA oligonucleotide against *stat3* (*stat3*-1, *stat3*-2 and *stat3*-3) were designed according to the sequence of human *stat3* gene (Genebank: NM003150). These oligonucleotides contain a sense strand with 19 nucleotides followed by a short spacer (TTCAAGAGA). The reverse complement of the sense strand has five thymines as a stop signal of RNA polymerase III transcription. The sequences of oligonucleotides were as follows:

*stat3*-1:5′ - GATTGACCTAGAGAC-CCACTTCAAGAGAGTGGGTCTCTAGGT-CAATCTTTTT-3′ (forward) and 5′-AATTAAAAAG ATTGACCTAGAGACCCACTCTCTTGAAGTG GTCTCTAGGTCAATCGGCC-3′ (reverse); *stat3*-2:5′-GAGTCGAATGTTCTCTATCTTCAAGAGAGATAGAGAACATTCGACTCTTTTT-3′ (forward); and 5′-AATTAAAAAGAG TCGAATGTTCTCTATCTCTCTTGAAGATAGA GAACATTCGACTCTGGCC-3′ (reverse); *stat3*-3:5′-GCAGCAGCTGAACAACATGCATGTTCAAGAGACATGTTGTTCAGCTGCTGCTTTTT-3′ (forward), and 5′-AATTAAAAAGCAGCAGCTGAACAACATGTCTCTTGAACATGTTGTTCAG CTGCTGCTGCGGCC-3′ (reverse).

These Oligos were annealed in the annealing buffer (100 mM K-actate, 30 mM HEPES-KOH [pH 7.4], 2 mM Mg-acetate, and the mixture was incubated at 90°C for 3 min and then at 37°C for 1 h) and cloned into the ***Hind*** III-***BamH*** I sites of pSliencer 2.1-U6 vectors^18^ which can express hairpin siRNAs under the control of U6 promoter. The pSilencer2.1-U6 was linearized with the ***Hind*** III-***BamH*** I restriction enzymes. The products with the double-stranded **s**tructure were used to form a recombinant plasmid with T4 DNA ligase followed by annealing. A negative control scrambled siRNA (Ambion, USA), which has no evident homology to mouse or human *stat3* sequences, was also designed aiming to exclude non-specificity.

### Cell culture and transfection

Human ovarian cancer cells (SKOV3 cells) were grown in Iscove’s Modified Dulbecco’s Medium (IMDM) (Invitrogen, USA) containing 10% fetal bovine serum (FBS). When the cell confluence reached 80∼90%, SKOV3 cells were washed three times with serum-free medium and divided into three groups: mock group, pSi-scramble siRNA group and pSi-*stat3* siRNA group. LipofectAMINE 2000 (Invitrogen, USA) was used in the cell transfection, and enhanced green fluorescent protein vector (pEGFP; BD Clontech, Inc; USA) was cotransfected with either pSilencer 2.1-U6-*stat3* siRNAs or pSi-lencer 2.1-U6-scrambled siRNA at a volume ratio of 1:20 to label the positive transfected cells. The transfection was performed for 5∼20 h and then the medium was refreshed with medium containing 10% FBS followed by lysis for 24∼72 h after the transfection.

### mRNA quantification

Total RNA was extracted from tissues with Trizol (Invitrogen, USA) following the manufacturer’s instructions. For Northern blot analysis, 20 μg of total RNA were separated by 1.2% agarose-formaldehyde gel, and blotted onto Hybridization-***N*** membranes (Amersham Pharmacia Biotech, USA). Hybridization was performed using the express Hyb buffer (BD Clontech, USA) with ^32^P-labeled cDNA of Survivin and actin as probes. Blots were exposed to Kodak MS film and then quantitated using a Molecular Dynamics Phosphorlmager.

### Assay of growth and cell cycle in vitro

SKOV3 cells were incubated in 96-well plates. The cell proliferation was determined by 3-(4,5-dimethylthiazol-2-y1)-2,5-diphenyltetrazolium bromide (MTT; Sigma, USA) assay and the number of viable cells were counted with a hemocytometer at 72 h after the transfection. The absorbance at 570 nm (A_570_) was determined with a microplate reader. The growth inhibition rate was calculated according to the following formula:

$$\text{Growth}\;\text{inhibition}\;\text{rate}\;(\%)=\left[\left({\text{A}}_{570\text{c}}-{\text{A}}_{570\text{e}}\right)/{\text{A}}_{570\text{c}}\right]\times 100\%$$

A_570c_: A_570_ in control group; A_570e_: A_570_ in experimental group

For the assay of cell cycle, SKOV3 cells were transfected with siRNA-stat3s or siRNA-scrambled vectors. After 72 h of transfection, these cells were collected, washed with phosphate buffered saline (PBS) containing 4 mmol/L ethylenediaminetetraacetic acid (EDTA), and fixed in cold 70% ethanol followed by centrifugation. The supernatant was removed and cells were washed once with PBS containing 4 mmol/L EDTA. Cells were then re-suspended in PBS containing 4 mmol/L EDTA, 20 ml/L of propidium iodide (Sigma, USA), 0.2% Triton X-100, and 40 mg/L RNase A followed by incubation for at least 30 min at 4°C. The cell cycle was detected with a flow cytometer (FACScan, Becton Dickinson, USA) followed by the analysis with Cell Quest software. For the determination of apoptotic cells, 95 μl of floating cells were mixed with 0.1% AO/EB (acridine orange/ethidium bromide [Sigma, USA]) followed by the observation under a microscope.

### In vivo growth of cancer cells

SKOV3 cells (3×10^6^) were subcutaneously inoculated into the back of 18 nude mice. The tumor volume (m_1_^2^×m_2_×0.5236, where m**_1_** represents the short axis and m**_2_** the longer axis) was measured every 2∼3 d until the tumors reached about 50.56±36.45 mm^3^ (by day 12) in volume. Then, these nude mice were randomized into three groups (n=6): (1) mock transfection group (PBS buffer alone); (2) scrambled vector group (20 μg/mouse); (3) pSilencer2.1-U6-*stat3*-siRNA group (20 μg/mouse). The plasmids were diluted in 50 μl of PBS and injected percutaneously into the tumors by using a syringe with a 27-gauge needle. Immediately after the injection, tumors were pulsed with an electroporation generator (ECM 830, BTX, USA). Pulses were delivered at a frequency of 1/sec and 150 v/cm for 50 ms. This process was repeated once a week, mice were sacrificed on day 33. The tumor volume was determined and then tumors were collected for H&E staining and terminal deoxynucleotidy1 transferase-mediated nick end labeling (TUNEL) assay.

### HE staining and TUNEL assay

Serial sections of tumor tissues were fixed in formalin, stained with H&E, and processed for the routine histological examination. TUNEL assay was done by using the In Situ Cell Death Detection Kit (Roche, Switzerland). Paraffin-embedded tissues were cut into 3-μm sections, deparaffinized and hydrated according to the standard protocol. After the incubation with proteinase K (200 μg/ml) for 30 min at 21°C, the TUNEL reaction mixture containing bromodeoxyuridine triphosphate, terminal deoxynucleotidy1 transferase, and reaction buffer was added onto the sections which were incubated in a humidified chamber for 60 s at 37°C, followed by washing and incubation with a FITC-labeled anti-bromodeoxyuridine monoclonal antibody for 30 min at room temperature. The reaction was visualized under a fluorescence microscope. TUNEL-positive cells present green fluorescence. The apoptotic index was calculated as follows: apoptotic index = (number of apoptotic cells/ number of total cells) ×100%.

### Statistical analysis

The statistical analysis was done with SPSS 13.0 statistic software package. The significance of the differences between various samples was determined using the student’s two-tailed t test. The comparisons between medians were performed with the two-tailed Mann-Whitney test. A value of *P*<0.05 was considered statistically significant.

## Results

### Stat3 is over-expressed in ovarian cancer cells and ovarian cancer tissues

**FIGURE 1:**
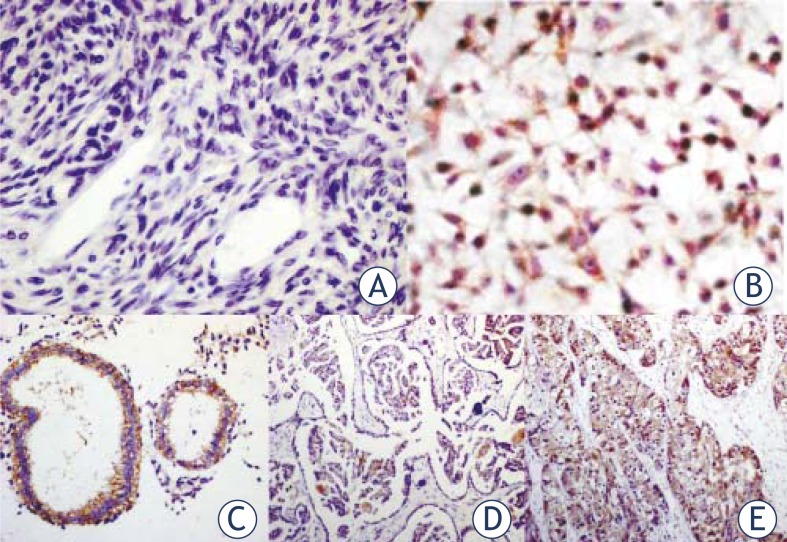
Expression of STAT3 in SKOV3 cells, ovarian cancer tissues, and normal ovarian tissue. A: normal ovarian tissue; B: SKOV3 cells; C: ovrian serous cystadenocarcinoma; D: ovarian mucinous cysadencarcinoma; E: clear-cell ovarian carcinoma.

The STAT3 expression in normal ovarian tissue (n=20), ovarian cancer tissues (n=25) and ovarian cancer cells (SKOV3 cells) was determined by using immunohistochemistry. Normal ovarian tissues showed low to moderate expression of Stat3. In contrast, primary ovarian cancer tissues and ovarian cancer cells had moderate to high expression of STAT3 (Figure1). Immunohistochemistry revealed that *stat3* expressions in 85% of ovarian cancers were classified as ++ to +++, which were significantly different (*P*<0.001) from that in normal ovarian tissues (+).

### Stat3-specific siRNA specifically reduces Stat3 expression in SKOV3 cells

**FIGURE 2:**
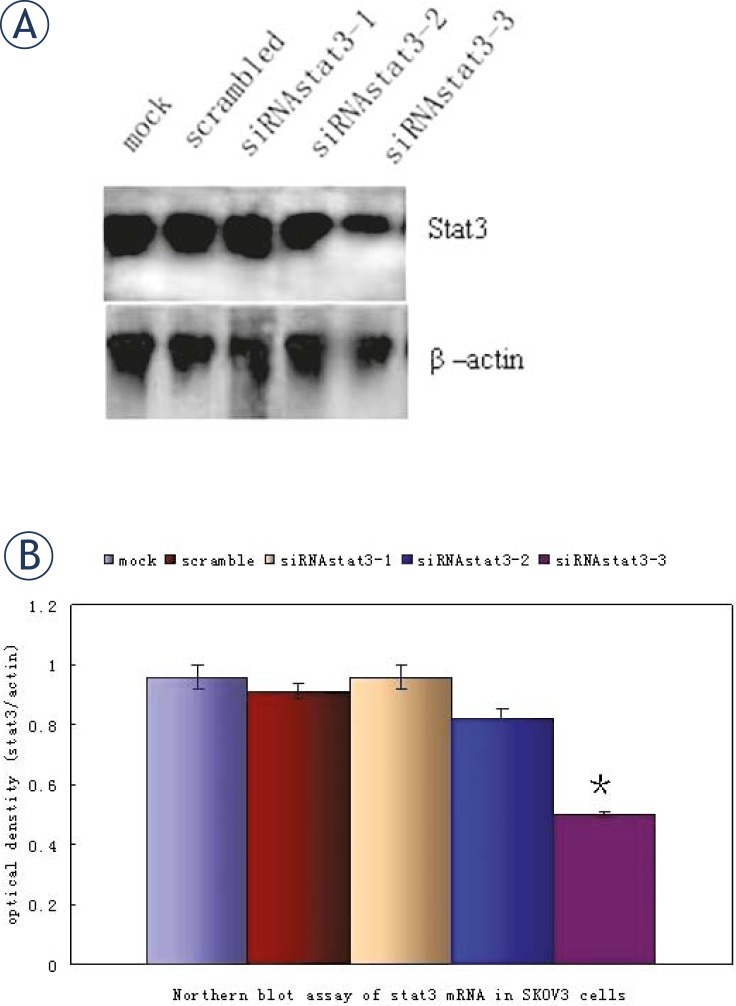
Northern blot assay of *stat3* mRNA in human ovarian cancer cell line. A: SKOV3 cells were treated with either 2 μg of pSliencer 2.1-U6 vector *stat3* siRNAs or scrambled siRNA vector for 72 h. B: Quantification of *stat3* mRNA expression from three separate experiments, which was normalized by that of β-actin. (*P<0.05 vs scrambled siRNA group).

**FIGURE 3:**
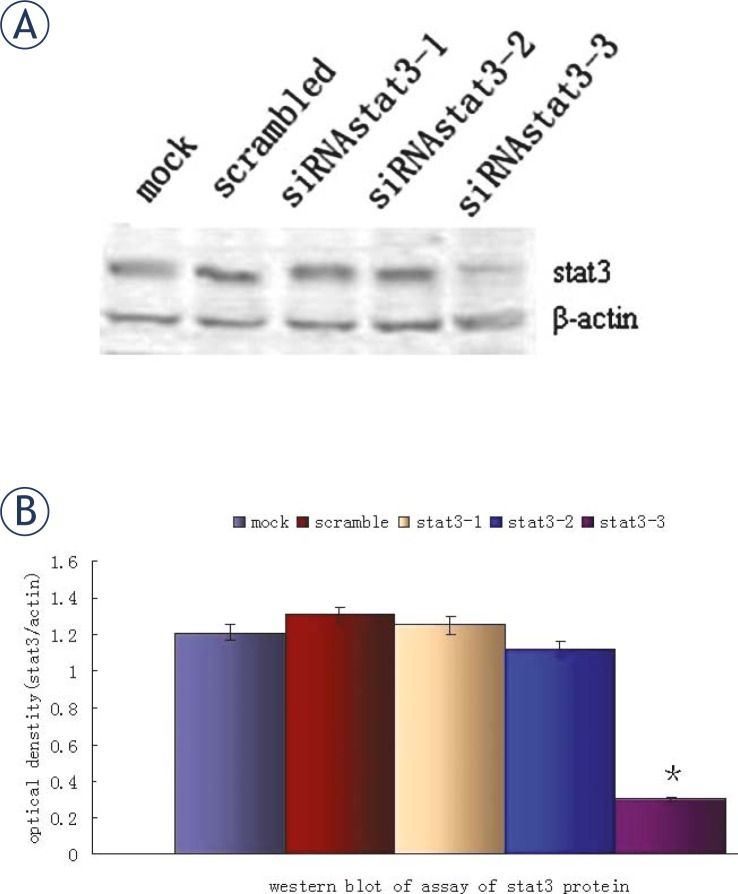
Western blot assay of STAT3 protein in human ovarian cancer cells. A: SKOV3 cells were treated with either 2 μg of pSliencer 2.1-U6 vector *stat3* siRNA or scrambled siRNA vector for 72 h. B: Quantification of STAT3 protein from three separate experiments, which was normalized by that of β-actin. (*P<0.05 vs controls)

Previous studies have provided strong evidence that siRNA specific to *stat3* gene can significantly suppress STAT3 protein expression.^18^,^19^,^21^ To determine if STAT3 expression in the ovarian cancer can be suppressed via the gene-silencing effect of vector-based RNAi, the siRNAs targeting different sites of *stat3* gene were designed previously which inhibited STAT3 expression significantly in prostate cancer cells.^22^ In the present study, SKOV3 cells were also transfected with siRNA and results showed only siRNA targeting *stat3*-3 could decrease the STAT3 expression significantly at both mRNA (Figure 2) and protein (Figure 3) levels when compared with scambled-siRNA. The siRNA targeting *stat3*-1 and *stat3*-2 had no significantly inhibitory effects on the mRNA and protein expressions of *stat3*.

### Inhibition of stat3 suppresses expressions of BcL-2, cyclin D1, and c-Myc in SKOV3 cells

**FIGURE 4:**
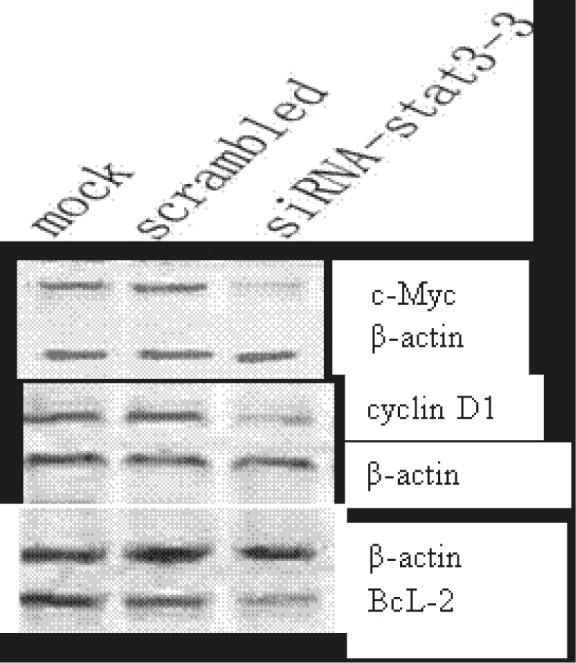
Western blot assay of c-Myc, cyclin D1 and BcL-2 in SKOV3 cells treated with either 2 μg of pSliencer 2.1-U6 *stat3-3* or scrambled siRNA vector for 72 h.

Constitutive activation of STAT3 induces the expressions of several genes including anti-apoptotic gene BcL-2, cyclin D1 and c-Myc which promote cell division. In order to determine whether these genes were involved in the Stat3-mediated apoptosis blocking in SKOV3 cells, Western blot assay was performed. Figure 4 shows that the BcL-2, cyclin D1 and c-Myc were highly expressed in the scrambled-siRNA transfected cells, whereas the expression levels of these proteins were significantly decreased after the transfection with siRNA-*stat3*.

### Stat3 siRNA inhibits in vitro growth and survival, induces apoptosis of SKOV3 cells and arrests SKOV3 cells in G1 phase

**FIGURE 5:**
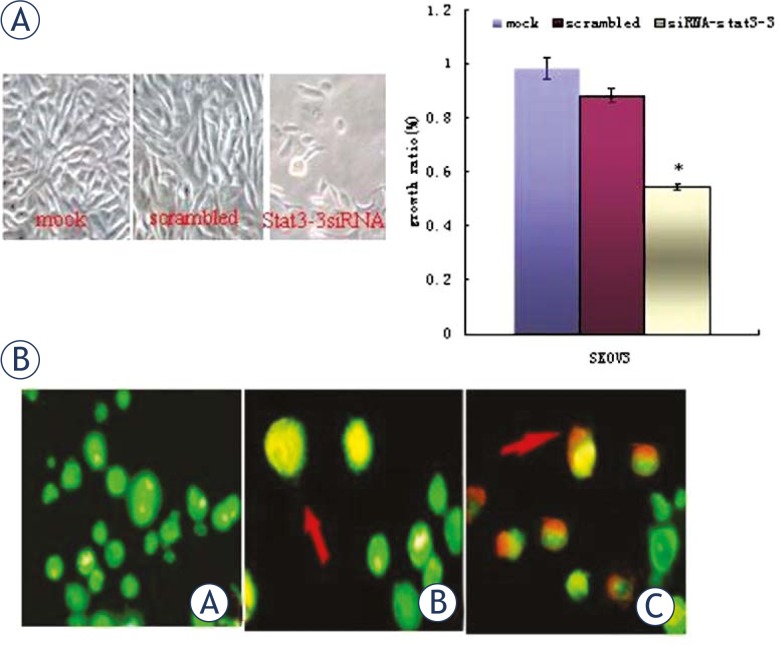
siRNA-Stat3-3 transfection significantly inhibited the growth of SKOV3 cells and induced apoptosis (A) transfection with *stat3*-3 siRNA for 72 h inhibited the growth of cancer cells (×400). B: Representative fluoromicrographs of apoptosis detected by AO/EB assay in control and *stat3*-3-siRNA–treated cells. C: SKOV3 cells (D) red arrow indicates early apoptotic cells; (E) red arrow indicates late apoptotic cells.

In order to determine whether *stat3*-siRNA had inhibitory effects on the growth of SKOV3 cells, MTT assay was conducted to determine the cell proliferation. Results showed the cells transfected with siRNA-*stat3* became less confluent and some cells were rounded and detached from the plates, when compared with cells transfected with scrambled vectors (Figure 5). AO/EB staining (nucleus condensation) was performed to detect the apoptotic cells. Results showed both early apoptotic and late apoptotic SKVO3 cells were seen in cells transfected with siRNA-*stat3*-3 (Figure 5). Flow cytometry revealed the apoptosis rate of siRNA-*stat3*-3 transfected cells was significantly higher than that of scrambled siRNA transfected cells (Table 1).

### In vivo anti-tumor activity of stat3 siRNA

**FIGURE 6:**
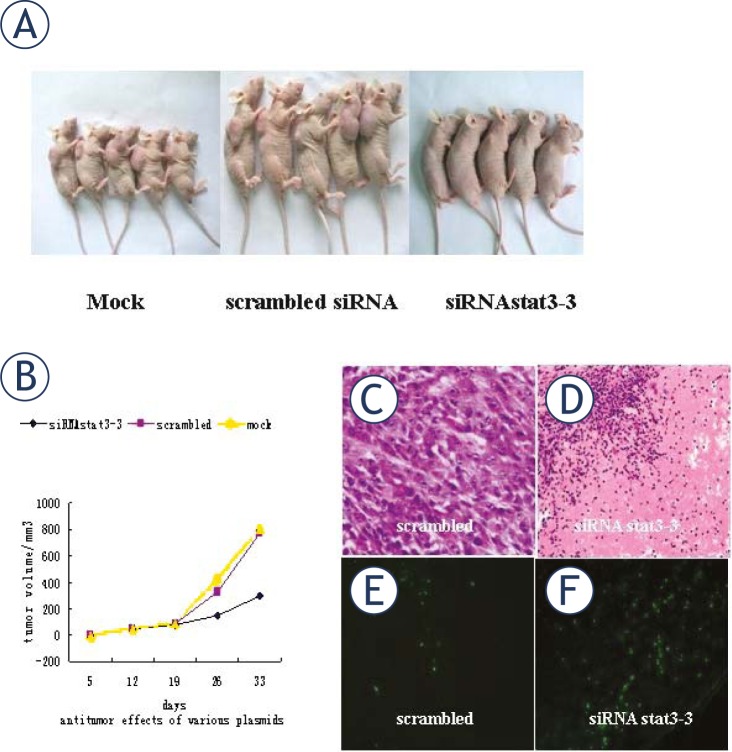
Intratumoral electroinjection of Stat3-3 siRNA resulted in significant inhibition of cancer growth and induced apoptosis of cancer cells *in vivo*. (A) Mice treated with scrambled vectors had visible cancers, whereas mice treated with 20 μg of *stat3*-3siRNA vectors had reduced tumor volumes. (B) Growth curves of cancer cells treated with *stat3*-3 siRNA. Mice were inoculated subcutaneously with SKOV3 cell. On day 12, the mean volume of palpable cancers reached 50.56±36.45 mm^3^ (n=6) at the sites of injection. Then, these mice were injected intratumorally with buffer, *stat3*-3 siRNA or scrambled siRNA. Injection was repeated on days 19 and 26, and the tumor sizes were determined on days 0, 5, 12, 19, 26 and 33. (Mean±SEM. n=6, *P*<0.01). (C, D) HE staining (100×). (E, F) TUNEL staining (200×).

In order to evaluate the effects of *stat3*-siRNA on the ovarian cancer growth *in vivo*, *stat3*-siRNA was injected into ovarian cancer bearing nude mice. Mice were subcutaneously inoculated with 3×10^6^ SKOV3 cells and the tumors were palpable at the sites of injection on day 12. Then, the mice were intra-tumorally injected with PBS, scrambled-siRNA or siRNA-*stat3*-3. This process was repeated at days 19 and 26, and animals were killed on day 33. The mean tumor volume in mice treated with scrambled siRNA was 767.65±100.23 mm^3^, and that in mice treated with siRNA *stat3*-3 was 298.23±19.89 mm^3^, showing the significant difference in tumor volume between these two groups. However, there was no marked difference between mice transfected with scrambled-siRNA and those treated with buffer (*P*>0.05) (Figure 6). To determine the mechanism of suppressed cancer growth *in vivo*, tumors were collected for H&E staining and TUNEL staining. Results showed siRNA-*stat3*-3 transfected cells underwent massive apoptosis with sparsely dispersed chromatin and necrotic tissue (Figure 6) and several TUNEL-positive cells or cell clusters (Figure 6), which was seldom found in the other two groups. These findings demonstrate the intra-tumoral administration of *stat3*-3 siRNA exerts potent suppressive effects on the cancer growth.

## Discussion

Under physiological conditions, STAT3 activation is a rapid process and activated STAT3 has short half life. The study has demonstrated *stat3* plays an important role in maintaining physiological functions of cells. STAT3 is a critical element in the epidermal growth factor receptor (EGFR), interleukin-6 (IL-6)/Janus kinase (JAK) and other carcinogenic tyrosine kinase signaling pathways. Evidence reveals STAT3 is expressed in a variety of human malignancies, including leukemia, multiple myeloma, multiple melanoma, squamous cell carcinoma of the head and neck (SCCHN), breast cancer, prostate cancer, ovarian cancer and lung cancer. The activated *stat3* plays crucial roles in the occurrence, growth, apoptosis inhibition of cancer cells.^23^–^25^ Rosen *et al.*^26^ indicated the persistent activation of STAT3 signal transduction was very important in the occurrence of ovarian cancer and could promote the cell proliferation resulting in occurrence of cancers. The persistent activation of STAT3 helps the cancer development, and a high expression of STAT3 also indicates a poor prognosis of the cancer. Therefore, *stat3* can be used as a novel target for the anti-cancer therapy and may become a novel cancer marker.

Our results showed the STAT3 expression was significantly increased in human ovarian cancer when compared with that in normal ovary tissues. Among the three pairs of double stranded siRNA oligonucleotide against *stat3*, only siRNA targeting *stat3*-3 could markedly inhibit the STAT3 expression in SKOV3 cells demonstrated by Western Blot and Northern Blot. Then, siRNA-*stat3*-3 was used to transfect SKOV3 cells for further experiments. Results revealed transfection with siRNA-*stat3* significantly decreased the expressions of BcL-2, cyclin D1 and c-Myc in SKOV3 cells. In addition, *stat3* siRNA inhibits the *in vitro* growth and survival, induces apoptosis of SKOV3 cells and arrests SKOV_3_ cells in G1 phase. Furthermore, the anti-tumor effects of *stat3* siRNA were also confirmed in ovarian cancer bearing nude mice. Taken together, these findings demonstrated the *in vitro* and *in vivo* anti-tumor effects of *stat3* siRNA.

Our results were similar to that of Cai *et al*.^27^ in which they also found the anti-tumor effects of *stat3* siRNA *in vitro* and *in vivo*. However, in the present study, we designed three pairs of double stranded siRNA oligonucleotide against *stat3*, and the most effective siRNA in the inhibition of STAT3 expression was selected. In addition, the suppressed expression of *stat3* was confirmed by Western Blot and Northern Blot at protein and mRNA levels, respectively. In the *in vivo* experiment, we detected not only changes of the tumor size but also the apoptosis of cancer cells in the tumors. In 2008, Huang *et al.* applied shRNA to knockdown *stat3* expression in CAOV3 ovarian cancer cell line demonstrating similar results.^28^ Their results suggest RNA interference is an effective and feasible strategy to down-regulate *stat3* expression in the treatment of ovarian cancer.

Bcl-2 is the founding member of the Bcl-2 family of apoptosis regulator proteins. The Bcl-2 gene has been implicated in a number of cancers. It is also thought to be involved in resistance to the conventional cancer treatment.^29^ Cyclin-D1, in humans is encoded by the CCND1 gene, that belongs to the highly conserved cyclin family whose members are characterized by a dramatic periodicity in protein abundance throughout the cell cycle. Mutations, amplification and over-expression of CCND1 gene, which alters the cell cycle progression, are observed frequently in a variety of tumors and may contribute to tumorigenesis.^30^ c-Myc protein is a transcription factor that activates the expression of a great number of genes through binding on consensus sequences and recruiting histone acetyltransferases (HATs). Myc gene is a very strong proto-oncogene and very often found to be up-regulated in many types of cancers.^31^ Based on the results of the present study, we speculated that the anti-tumor effects of siRNA targeting *stat3* may be related to the cell cycle arrest and promotion of the apoptosis of cancer cells.

In summary, our results indicate *stat3* siRNA treatment can significantly inhibit the growth of ovarian cancer cells and promote their apoptosis. Thus, we postulate that STAT3 can be used as a therapeutic target for ovarian cancer patients and RNA interference with siRNA targeting *stat3* may become an effective strategy for the treatment of ovarian cancer.

## Figures and Tables

**TABLE 1:** Apoptosis of SKOV3 cells after transfection with siRNA-*stat3-3* (n=3) ±sd

**Group**	**Apoptosis rate (%)**
Mock	0.02 ± 0.00
Scrambed siRNA	2.49 ± 1.54
siRNA-Stat3-3	16.6 ± 3.43*

* P <0.05 vs. mock and scrambled groups.
